# FGA-Corn: an integrated system for precision pesticide application in center leaf areas using deep learning vision

**DOI:** 10.3389/fpls.2025.1571228

**Published:** 2025-07-08

**Authors:** Zhongqiang Song, Wenqiang Li, Xuehang Song, Shun Li

**Affiliations:** ^1^ School of Physics and Electronic Information, Weifang University, Weifang, Shandong, China; ^2^ College of Science, Henan Agricultural University, Zhengzhou, Henan, China

**Keywords:** precision agriculture, center leaf detection, embedded device deployment, realtime detection, precision pesticide delivery system

## Abstract

**Introduction:**

In corn pest and disease prevention, traditional blanket pesticide spraying has led to significant pesticide waste and environmental pollution. To address this challenge, research into precision agricultural equipment based on computer vision has become a hotspot.

**Methods:**

In this study, an integrated system named the FGA-Corn system is investigated for precision pesticide application, which consists of three important parts. The first part is the Front Camera Rear Funnel (FCRF) mechanical structure for efficient pesticide application. The second part is the Agri Spray Decision System (ASDS) algorithm, which is developed for post-processing the YOLO detection results, driving the funnel motor to enable precise pesticide delivery and facilitate real-time targeted application in specific crop areas. The third part is the GMA-YOLOv8 detection algorithm for center leaf areas. Building on the YOLOv8n framework, a more efficient GHG2S backbone generated by HGNetV2 enhanced with GhostConv and SimAM is proposed for feature extraction. The CM module integrated with Mixed Local Channel Attention is used for multi-scale feature fusion. An Auxiliary Head utilizing deep supervision is employed for improved assistive training.

**Results and discussion:**

Experimental results on both the D1 and D2 datasets demonstrate the effectiveness and generalization ability, with mAP@0.5 scores of 94.5% (+1.6%) and 90.1% (+1.8%), respectively. The system achieves a 23.3% reduction in model size and a computational complexity of 6.8 GFLOPs. Field experiments verify the effectiveness of the system, showing a detection accuracy of 91.3 ± 1.9% for center leaves, a pesticide delivery rate of 84.1 ± 3.3%, and a delivery precision of 92.2 ± 2.9%. This research not only achieves an efficient and accurate corn precision spraying program but also offers new insights and technological advances for intelligent agricultural machinery.

## 1 Introduction

Corn, as a key global food and chemical raw material, had a production of 45.507 billion bushels in 2022-2023 (World of Corn, 2023). However, annual production losses of 20% to 40% were attributed to pests and diseases (Kamilaris and Prenafeta-Boldú, 2018). Traditional blanket pesticide spraying, including plant protection drones, is a significant method to increase production and reduce costs (Tudi et al., 2021). Despite that, the indiscriminate spraying method could lead pesticide waste. Therefore, precision agriculture has gradually become a research hotspot in recent years, which utilized sensors and radar, especially the computer vision technology for the localization of individual plants (Salazar-Gomez et al., 2021). Based on the detailed location information, researchers can achieve effective regional management and investment to improve resource utilization and food production efficiency (Fountas et al., 2020).

With the continuous advancements in deep learning and convolutional neural network (CNN) technologies, the accuracy of machine vision has significantly improved, promoting its application in precision agriculture. It was reported that the accuracy of strawberry detection and grapevine key point detection could achieve to mAP of 82.44% (Zhang et al., 2022) and AP of 89.7% (Chen et al., 2024). In corn cultivation, existing research has primarily focused on using computer vision for pest and disease identification (Du et al., 2022; Divyanth et al., 2023; Antolínez García and Cáceres Campana, 2023; Mota-Delfin et al., 2022), weed recognition (Jiang et al., 2020), and real-time growth monitoring through phenotyping studies (Guan et al., 2024). In agricultural intelligent precision spraying research, recent studies demonstrated various successful implementations. Drones with cameras and image analysis capabilities for precise crop protection (Cisternas et al., 2020). TF Lite models deployed on Raspberry Pi-equipped drones for autonomous spraying decisions (Singh et al., 2024). Notable achievements include multi-object tracking for avoiding duplicate spraying (Hu et al., 2024), high-precision strawberry spraying using ONNX-quantized YOLOv3 with 97% accuracy (El Amraoui et al., 2024), and weed-targeted herbicide application achieving over 90% effectiveness in both controlled and field conditions (Upadhyay et al., 2024).

While high-precision recognition models provide technical support for precision agriculture, their complexity and numerous parameters present deployment challenges. To address these issues, researchers have focused on designing lightweight network architectures for resource-constrained devices. Key developments include efficient depthwise separable convolutions and subsequent versions of MobileNet (Howard, 2017; Sandler et al., 2018; Howard et al., 2019). Other advancements involve the cost-effective feature map generation of GhostNet (Han et al., 2020). Improvements were also made to the DFC attention mechanism of GhostNetV2 (Tang et al., 2022). Furthermore, the HGNetV2 backbone of RT DETR (Zhao et al., 2024) contributed to real-time detection. Recently, the effectiveness of channel or spatial attention mechanisms in generating clearer and more refined feature representations has been validated (Zhang et al., 2023). Parameter-free attention mechanisms like SimAM (Yang et al., 2021) and MLCA (Wan et al., 2023) have demonstrated enhanced model efficiency and generalization capability for embedded applications.

Therefore, the feasibility of achieving high precision through 2D machine vision and simplified mechanical structures was explored, highlighting its importance. Compared to complex 3D systems, 2D vision was noted for its lower hardware costs and simpler structures, as well as reduced dependence on environmental conditions. However, this shift imposed higher demands on algorithm accuracy and mechanical design. The research focused on achieving high-precision pesticide application using target detection information without reliance on complex location data.

In summary, the feasibility of achieving high precision through 2D machine vision and simplified mechanical structures was explored. In this study, an embedded delivery system for row-planted corn was developed, providing hardware support for precision pesticide application in agriculture. First, a simple and efficient mechanical device named FCRF was designed based on the structural characteristics of row-planted corn. Second, an ASDS algorithm uses post-processing of detection results to coordinate visual models with mechanical structures to accurately apply pesticides. Third, a lightweight 2D vision algorithms were investigated based on YOLOv8 for high-precision detection and deployment. This design shifts pesticide application from traditional blanket spraying to center leaf areas of corn, which fundamentally supports automated farming and pest management, and promotes the development of precision agriculture.

## 2 Materials and methods

**Figure 1:**
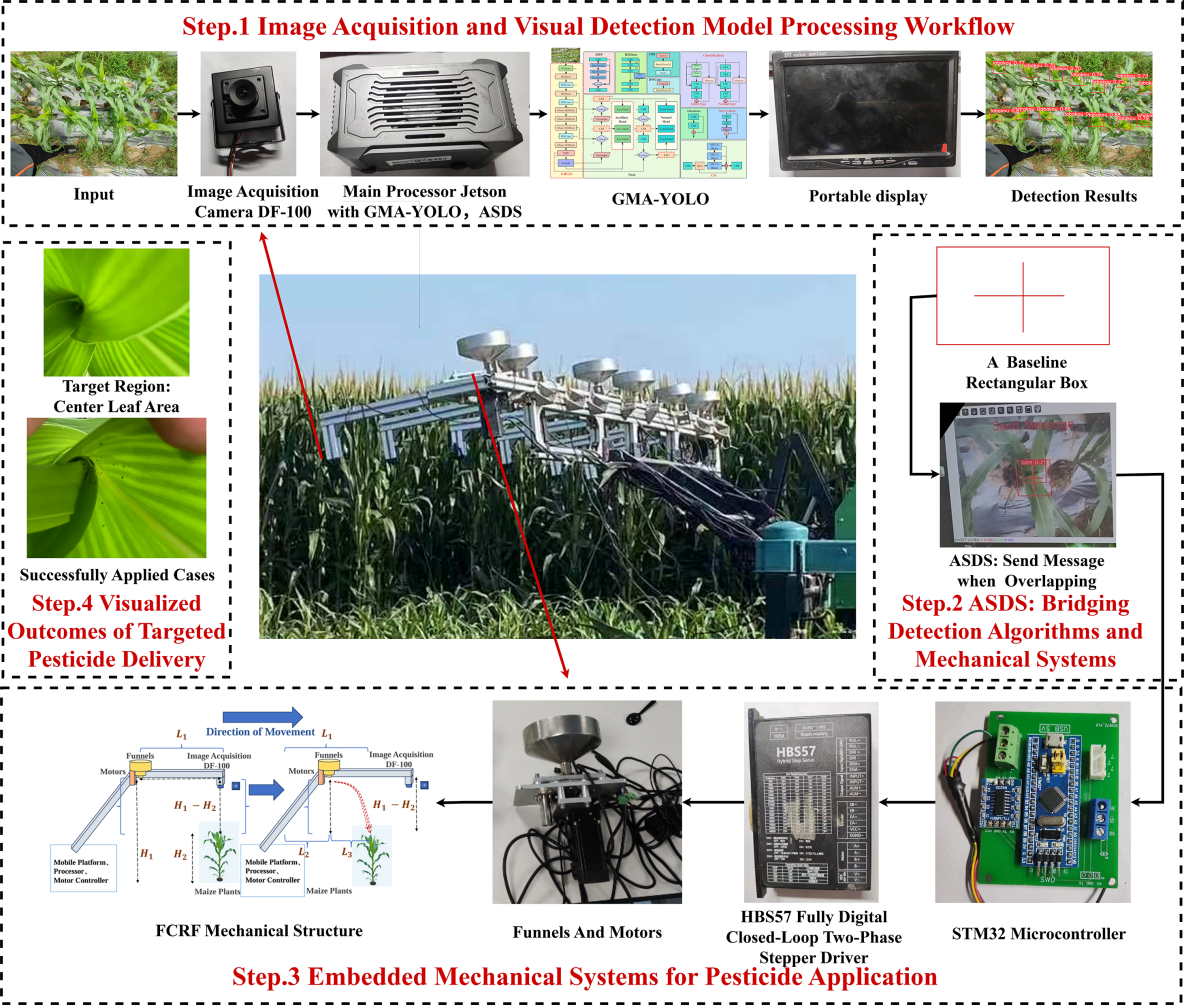
FGA-Corn workflow: from image acquisition to pesticide application on center leaf areas.

A system called FGA-Corn was developed for precise pesticide delivery during the corn growth stage. This system consists of an efficient mechanical structure (FCRF), an optimized deep learning object detection model (GMA-YOLOv8), and software algorithms (ASDS) for post-processing detection information. **Figure 1** illustrates the complete workflow, from camera image acquisition to precise pesticide application.

The system integrates image processing and deep learning algorithms for detecting center leaf areas, simultaneously actuating the mechanical structure for precise pesticide delivery. As shown in Step 1 of **Figure 1**, operation commences with the capture of corn imagery by a camera, which is then transmitted to an Nvidia Jetson Xavier NX edge device hosting the GMA-YOLO model for object detection. Subsequently, in Step 2, the ASDS algorithm refines these detection results through post-processing and simultaneously commands the integrated embedded and mechanical systems (Step 3) for targeted pesticide application. Finally, Step 4 of **Figure 1** depicts the visual comparison of the center leaf areas before and after pesticide application. This comprehensive system facilitates effective recognition of designated corn leaf regions and enables real-time precision targeting, thereby supporting informed and efficient pesticide application decisions.

### 2.1 FGA-corn precision pesticide application system

#### 2.1.1 System equipment and its parameters

The entire system hardware consisted of four main components: the decision layer, signal relay layer, execution layer, and image acquisition layer.

The core component of the decision layer was the Nvidia Jetson Xavier NX edge device (NVIDIA, Santa Clara, California, USA), on which the improved GMA-YOLOv8 algorithm was deployed. A key attribute of both Jetson Nano and Jetson Xavier NX is their integration of CPU and GPU in a heterogeneous architecture. This device was equipped with 384 NVIDIA Volta architecture GPUs (48 Tensor Cores) and a 6-core NVIDIA Carmel ARM v8.2 64-bit CPU, offering 128GB of storage and 8GB of memory, providing powerful AI computing capabilities.

The signal relay layer was composed of an STM32 development board (STMicroelectronics, Geneva, Switzerland), which was responsible for converting the signals generated by the ASDS algorithm. Additionally, the signal relay layer employed the HBS57 fully digital closed-loop two-phase stepper driver (Leadshine Technology, Dongguan, Guangdong, China) to precisely control the motor rotation of spraying funnel.

The execution layer included a funnel equipped with a rotating motor (PM60-10-ST/57CME30A, XINSONG, Shenyang, Liaoning, China), featuring a working current of 5.0 A and a torque of 3.0 N·m. By driving the HBS57 driver with an STM32 microcontroller, accurate control of the motor rotation was achieved. When the holes in the rotating motor briefly aligned with those in the funnel, the funnel opened, allowing pesticide granules to fall under the force of gravity.

The image acquisition layer utilized the DF-100 industrial camera (Jierui Weitong, Shenzhen, China), which featured a 2.8mm diagonal (1/2.7-inch) CMOS sensor with 2 million physical pixels. This camera captured plant information in real time. The image transmission frame rate was set to 30 frames per second, and the DF-100 communicated with the Jetson via USB 3.0, ensuring real-time data acquisition and storage.

The display and debugging layer included a portable display, a Bluetooth keyboard, and a mouse, allowing for real-time debugging of software algorithms during field operations.

Through the design and implementation of these components, along with their detailed device parameters, the FGA-Corn system was able to efficiently and accurately perform pesticide spraying on the center leaf areas of corn.

#### 2.1.2 Front camera rear funnel mechanical structure

**Figure 2:**
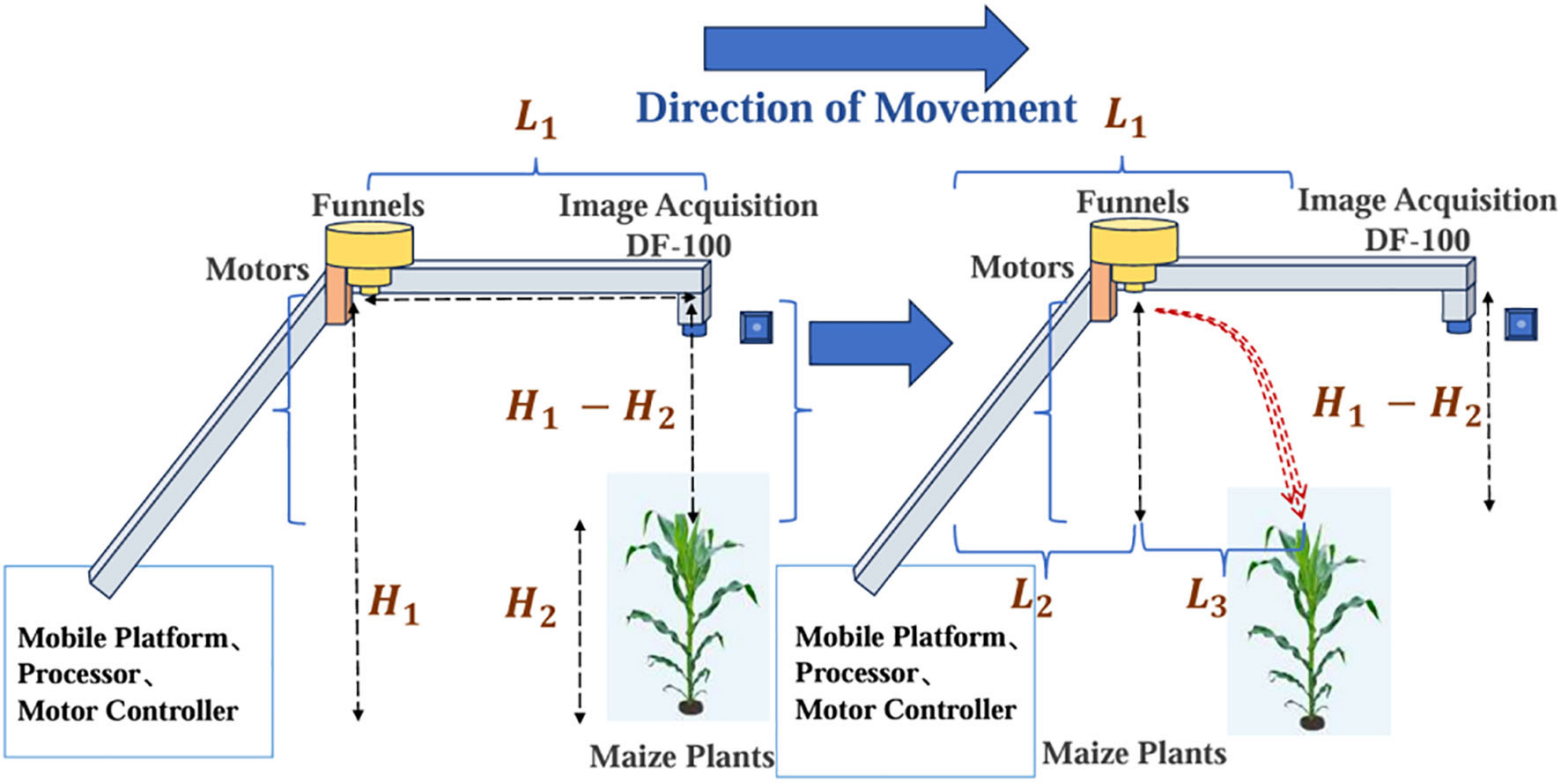
Front camera and rear funnel mechanical structure display diagram.

To achieve high-precision pesticide application in the center leaf areas of corn, a precision spraying system was developed that integrated visual perception and mechanical control, referred to as the FCRF. The core of this system is to place the camera in front of the spray funnel and ensure that the distance between the camera and the spray funnel is set to *L_1_*. As shown in **Figure 2**, the FCRF structure was highly modular, featuring a simple design and easy maintenance. It was cost-effective in both production and maintenance, making it scalable across various agricultural applications. During system operation, the spraying precision depended on the accurate synchronization of multiple timing parameters. First, from the moment the vision system captured the target area to the initiation of the spray funnel motor, there was an inherent signal processing and transmission delay, denoted as *T_1_* ​. Additionally, as pesticide particles fell from the height *H_1_* ​ to the target height *H_2_* ​ of the center leaf areas, a height difference Δ*H=H_1_-H_2_* existed. The time required for the particles to fall was represented by *T*_2_​. Assuming the mobile platform equipped with the system moved at a speed of *V_1_*, the time required to travel a distance of Length1 was *T*_3_​. For the system to achieve accurate spraying, the following relationship needed to be satisfied: *T_1_+T_2_=T_3_*. This implied that the total time for signal transmission and particle descent should equal the time the mobile platform took to reach the target position, ensuring that the pesticide was applied precisely at the intended location.

As shown in the **Figure 2**, during the signal processing delay *T_1_*, the mobile platform ran a distance of *L_2_*. Subsequently, the spray motor was activated, and the pesticide particles began to fall, covering a distance of *L_3_* before finally reaching the designated center leaf area of the corn. However, in real-world agricultural operations, the travel speed of platform *V_1_* could vary due to factors such as complex terrain and crop conditions, leading to dynamic changes in *T_3_*. To ensure the precise matching of *T_1_+T_2_=T_3_*, the system primarily achieved this through dynamic adjustment of the funnel motor rotational speed, while keeping path *L_1_* constant. The system could also dynamically adjust the additional delay *T_4_* in the signal transmission process. These adjustments compensated for any time deviations caused by changes in travel speed. The dynamic adjustment mechanism ensured that high accuracy in pesticide particle delivery was maintained during operations, thus enhancing agricultural efficiency and crop protection.

The mechanical structure was optimized for row-planted agricultural fields, where one delivery system corresponded to one row of plants. For corn fields specifically, the open structure of corn leaves allowed delivered solid granules to roll down toward the central target area, representing a critical characteristic that guided the FCRF design. This structure implemented an asynchronous operation between visual detection and pesticide application, utilizing and eliminating the delay between target detection and dispenser activation. The asynchronous operation demonstrated another significant advantage in preventing error accumulation, as detection and delivery were performed separately, enabling error correction in each delivery decision. In field operations, one FGA-Corn structure corresponded to one row of target plants, therefore, the pesticide delivery system was configured in a multi-row cascade arrangement to maximize coverage area and enhance operational efficiency.

#### 2.1.3 Agri spray decision system

**Figure 3:**
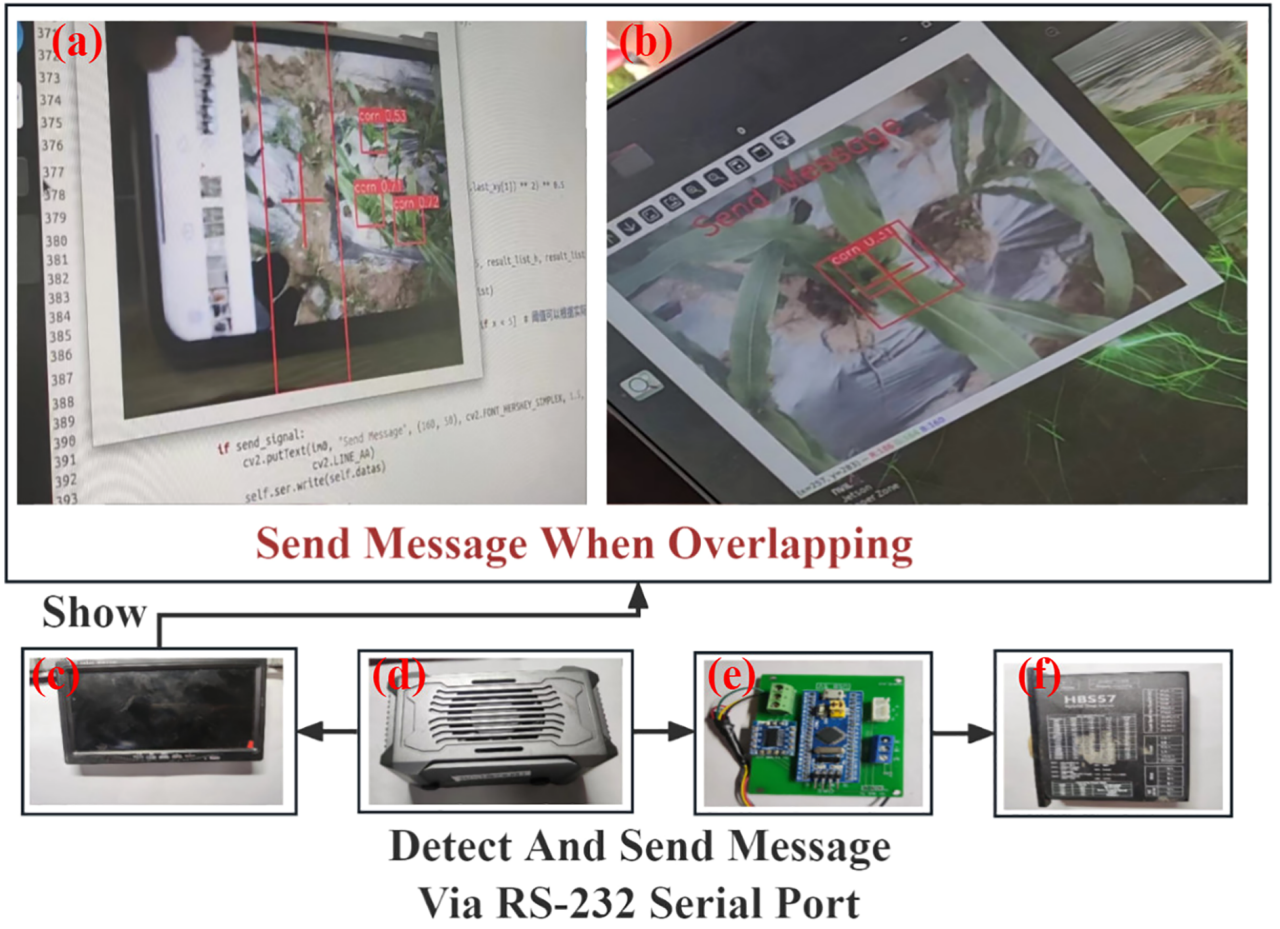
ASDS decision algorithm for YOLO post-processing in precision agriculture: **(a)** Post-processing visualization display window, **(b)** Signal transmission logic, **(c)** Portable display screen, **(d)** Jetson main control device, **(e)** STM32 microcontroller, **(f)** HBS57 fully digital closed-loop two-phase stepper driver.

The ASDS (Adaptive Spraying Decision System) is the core algorithm responsible for controlling precise pesticide spraying, with its specific process illustrated in **Figure 3**.

In this framework, this study defines post-processing algorithms and logic based on the YOLO (You Only Look Once) object detection system. Initially, a baseline rectangular box is manually defined on the detection window display page; this box contains a crosshair icon centered within it. When the detection system is operational, the YOLO algorithm processes images captured in real-time by the camera and performs post-processing logical judgments: if center leaf areas of corn are detected, the system evaluates whether this detected area overlaps with the predefined baseline rectangular box. Once the detection box meets the predefined overlap conditions with the baseline rectangular box, the system triggers the dosing decision and sends corresponding dosing instructions to the signal transmission layer. These instructions drive the spraying equipment via an STM32 microcontroller and an HBS57 fully digital closed-loop two-phase stepper driver, thereby achieving automated control of the detection algorithm and the dosing mechanical structure. To enhance the flexibility and adaptability of algorithm, the dimensions of this baseline rectangular box can be dynamically adjusted based on different growth stages of the corn and the actual growth conditions of the crops, thereby optimizing the precision of pesticide delivery. Further details are elaborated in **Algorithm 1**.

Algorithm 1 **Agri spray decision system.**

**Input**: Video stream V from camera
**Output**: Motor control signals via HBS50
//Initialize system components
1: **Initialize** RS232_PORT, BAUD_RATE
2: **Define** RECT_REGION = {x1, y1, x2, y2}//Detection zone coordinates
//Step 1. Main processing loop
3: **while** Video_Stream_Active **do**
4: frame ← GetNextFrame(V)
5: preprocessed_frame ← Preprocess(frame)
//Step 2. Object detection
6: detections ← GMA-YOLO_Detect(preprocessed_frame)
7: **for each** detection d in detections **do**
8: {conf, label, (x, y)} ← d
9: DisplayDetection(frame, d)
//Step 3. Check intersection with defined region
10: **if** IntersectsRegion(x, y, RECT_REGION) **then**
11: DisplayMessage(“send message”)
//Step 4. Serial communication protocol
12: signal_packet ← FormatSignal(x, y, conf)
13: **try**:
14: SendSerialData(RS232_PORT, signal_packet)
15: **catch** CommunicationError:
16: HandleError()
17: **end if**
18: **end for**
19: **end while**
20: **return** TRUE//Return success status after completion

Additionally, the parameters of the rotating motor in the execution layer (such as revolutions per minute) could be precisely controlled and adjusted through software code to accommodate various operational requirements and crop conditions.

Through this strategy that combined hardware adjustments with software control, a software-based pesticide delivery decision algorithm was developed, achieving precise and real-time delivery to the center leaf areas of corn. This approach not only simplifies manual operations but also improves pesticide application efficiency through intelligent image processing, reducing over spraying and enhancing the sustainability and economic benefits of the operation.

### 2.2 Data acquisition and preparation

This study documented and analyzed image data collected during pesticide application periods in the experimental fields of Henan Agricultural University, Zhengzhou. The data collection was conducted during the V6-V8 stage of corn, with images captured at different times (morning 7:00-9:00 AM, noon 11:00 AM-1:00 PM, and evening 4:00-6:00 PM) under sunny weather conditions. A total of 162 high-resolution images (4000×3000 pixels) were captured from an overhead view, with the camera positioned 0.5 to 1 meter above the corn. The center leaf areas were annotated with the LabelImg tool, and the dataset was split into training and test sets in an 8.2:1.8 ratio. To enhance the generalization ability of the model, a supplementary dataset (D2) containing 600 training images and 200 validation images was collected under identical conditions.

Motion blur augmentation was implemented to enhance detection capability under high-speed operation conditions, while random brightness adjustments (± 25%) and image rotation were applied to simulate natural lighting variations and different viewing angles. These methods expanded the initial dataset to 1,085 images and the D2 dataset to 2,400 images, strengthening the accuracy and robustness of the model in practical applications. Images of corn during pesticide application and the target detection area were shown in **Figure 3**.

### 2.3 GMA-YOLOv8 deep learning method

This study aimed to achieve real-time detection of the center leaf areas of corn during its growth stages. After comparing various network models, YOLOv8 was ultimately selected as the core model due to its exceptional accuracy, despite having room for improvement. To optimize performance, a series of comparative experiments were conducted, leading to the selection of HGNetV2 as the backbone. HGNetV2 employed a hierarchical lightweight feature extraction approach, effectively learning complex patterns across multiple scales and abstraction levels, thereby enhancing its capability to process complex image data.

Building on this foundation, the GHG2S backbone network was designed, integrating the lightweight attention mechanism SimAM to improve detection accuracy and speed. Research increasingly indicated that applying attention mechanisms for feature extraction and fusion could effectively mitigate the performance loss associated with model lightweighting. Parameter-free attention mechanisms like SimAM and MLCA enhanced feature focus while maintaining high computational efficiency and model simplicity, making them ideal for agricultural edge devices and embedded systems.

**Figure 4:**
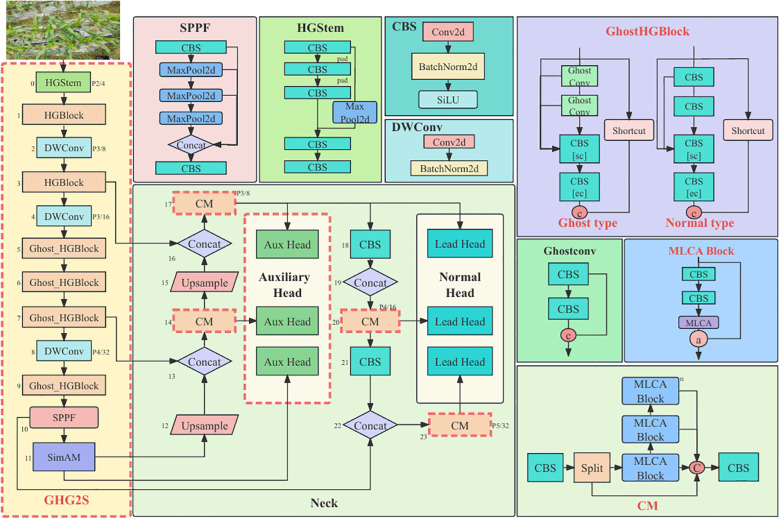
Improved model overview display.

To effectively integrate feature information, the Mixed Local Channel Attention (MLCA) mechanism was introduced, and the CM (C2f-MLCA) was proposed. Additionally, an Auxiliary Head was incorporated for supplementary feature supervision to assist in training, achieving high-precision identification of corn leaf regions. Based on these designs and improvements, GMA-YOLOv8 maintained rapid detection speed while ensuring accuracy, meeting the demands of edge computing. The architecture of the improved model is illustrated in **Figure 4**.

#### 2.3.1 Standard YOLOv8 model

YOLOv8 (You Only Look Once), introduced by Ultralytics, represented a significant advancement over YOLOv5, enhancing both performance and versatility (Bochkovskiy et al., 2020; Redmon et al., 2016; Redmon and Farhadi, 2017; Redmon, 2018; Wang et al, 2023). The algorithm efficiency was improved, and new features were introduced to enhance applicability. The model structure included an input module that processed images using grayscale padding and data augmentation, a backbone network that combined CBS and SPPF for feature extraction—with the lightweight C2f module enhancing detail recognition—a neck network that integrated features of different scales using FPN and PAN structures, and a detection module that employed anchor-free heads and a new loss function for precise predictions. YOLOv8 also allowed parameter tuning for width (W), depth (D), and ratio (R) to create models of various sizes (N/S/M/L/X), supporting tasks like object detection and semantic segmentation across diverse industries. This study focused on optimizing the lightweight YOLOv8n model for edge computing deployment, aiming to provide a more efficient solution.

#### 2.3.2 Feature extraction backbone GHG2S

YOLOv8, developed by Ultralytics, employed techniques such as CBS, C2f, and SPPF for feature extraction in the backbone network. However, its complex network structure was not conducive to edge deployment. To address this issue, a new lightweight backbone network, named GHG2S, was designed as shown in **Figure 4**. The GHG2S structure was introduced through three improvement measures.

Firstly, a series of mainstream lightweight backbone networks was tested to select an appropriate structure. After these experiments, HGNetV2 was chosen to replace the original backbone network due to the significant reduction in model parameters achieved through its hierarchical feature extraction method. HGNetV2, serving as the backbone network of RT-DETR, utilized a hierarchical lightweight feature extraction method to learn complex patterns at multiple scales and abstraction levels, thereby enhancing the ability of this network to handle complex image data. Features were extracted through continuous convolution in the Stem layer, and integration of features at different levels was performed using the HGBlock in **Figure 4**, incorporating residual connections and specific convolution layers. The number of parameters was reduced, and the efficiency and expressiveness of high-level feature extraction were improved through DWConv downsampling. The outstanding performance of HGNetV2 in feature extraction was attributed to its excellent network architecture.

Despite its superior architecture, many repeated HGBlocks in HGNetV2 contained numerous ordinary convolutions that required substantial computational resources. Therefore, the second improvement involved replacing the convolutional layers in the HGBlock with GhostConv to further reduce the number of model parameters. The working details of GhostConv were as follows: the module first received the image and reduced the number of feature layers through non-linear convolution (CBS: Conv2D, BatchNormalization, SiLU), using fewer convolution kernels. Linear convolution (such as 3x3 or 5x5 kernels) was then applied to the feature map for feature mapping, followed by merging the results of these two steps. Mathematically, the input dimension was chw (input channel number, height, width), and the output dimension was nhw (output channel number, height, width). Assuming $\frac{n}{s}$ represented the number of output channels after the first transformation, Ghost convolution was shown to be significantly lower than regular convolution in terms of computation and parameter quantity after s transformations. This is particularly evident when comparing the sizes of the regular convolution kernel k and the linear transformation kernel d . As depicted in Equations 1 and 2, the efficiency of Ghost convolution is highlighted, demonstrating reduced computational load and fewer parameters required.


(1) $$R\_S=\frac{w'\cdot h'\cdot n\cdot c\cdot k^{2}}{w'\cdot h'\cdot \frac{n}{s}\cdot \left(c\cdot k^{2}\;+\;d^{2}\cdot \left(s-1\right)\right)}\approx s$$

(2) $$R\_C=\frac{s\cdot k^{2}}{k^{2}+\left(s-1\right)\cdot c\cdot d^{2}}\approx s$$

After the above two improvements, the model parameters of the backbone network were significantly reduced, but this slightly affected its ability to extract features, resulting in a reduction in accuracy. To compensate for this performance loss, the third improvement introduced the SimAM (Simultaneous Attention Module) attention mechanism at the end of the new backbone network. By focusing on channel and spatial information from a three-dimensional perspective, it enhanced the quality of feature extraction. SimAM was designed as a lightweight attention module to address the issues of traditional attention mechanisms, which required additional subnetworks (such as GAP+FC+ReLU+FC+Sigmoid) to generate weights and lacked flexibility. It did not add extra parameters and was capable of generating three-dimensional attention weights for feature maps that integrated spatial and channel dimensions.

SimAM utilized an energy optimization function based on neuroscience to evaluate the importance of neurons both concisely and efficiently, as illustrated in Equations 3. Most of its operations were based on this energy function, thereby reducing the need for structural adjustments. This method not only improved the portability of the module and flexibility but also enhanced its efficiency across various tasks. By defining the energy function and utilizing binary labels and regularization terms, denoted in Equation 4, SimAM accurately calculated the importance of neurons, as shown in Equations 5, 6. It simplified the process of obtaining analytical solutions, encapsulated in Equation 7, and effectively represented the statistical characteristics of neurons in the (H) and (W) dimensions, signified by Equation 8.


(3) $$e\_t\left(w\_t,b\_t,y\_t,x\_i\right)={\left(y\_t-\hat{t}\right)}^{2}+\frac{1}{M_{all}-1}\sum_{i=1}^{M_{all}-1}{\left(y\_o-\hat{x}\_i\right)}^{2}$$

(4) $$\begin{array}{c} e\_t\left(w\_t,b\_t,y\_t,x\_i\right)=\frac{1}{M_{all}-1}\sum_{i=1}^{M_{all}-1}{\left(-1-\left(w_{t}*x\_i+b\_t\right)\right)}^{2}\\ +{\left(1-\left(w\_t*t+b\_t\right)\right)}^{2}+\lambda w\_t^{2} \end{array}$$

(5) $$w\_t=\frac{-2\left(t-u\_t\right)}{{\left(t-\mu_{t}\right)}^{2}+2\sigma_{t}^{2}+2\lambda }$$

(6) $$b\_t=\frac{-1}{2}\left(t+u\_t\right)w\_t$$

(7) $$e\_t^{*}=\frac{4\left({\hat{\sigma }}^{2}+\lambda \right)}{{\left(t-\hat{\mu }\right)}^{2}+2{\hat{\sigma }}^{2}+2\lambda }$$

(8) $$\tilde{X}=\text{sigmoid}\left(\frac{1}{E}\right)⊙X\_o$$

Where w_t is the weight vector at time t, b_t is the bias term at time t, y_t is the true label, x_i is the input feature vector for the i-th sample; M_all is the total number of samples, λ is the regularization parameter, t is the target value, u_t is the mean of the target values, ${\hat{\sigma }}^{2}$ is the variance of the target values, E is the energy measure, X_o is the original input feature matrix.

#### 2.3.3 Neck Module CM and MLCABlock

YOLOv8n employed a C2f structure containing multiple bottleneck layers to extract features, aiming to improve the computational efficiency of the model. However, this structure had some limitations. First, C2f primarily enhanced the ability of this model to capture detailed features through convolution operations, but it exhibited limitations in the diversity and richness of feature representation. Second, C2f focused on the extraction of spatial features while overlooking the dynamic dependencies between channels. This imbalance limited performance in tasks requiring fine-grained recognition and classification.

**Figure 5:**
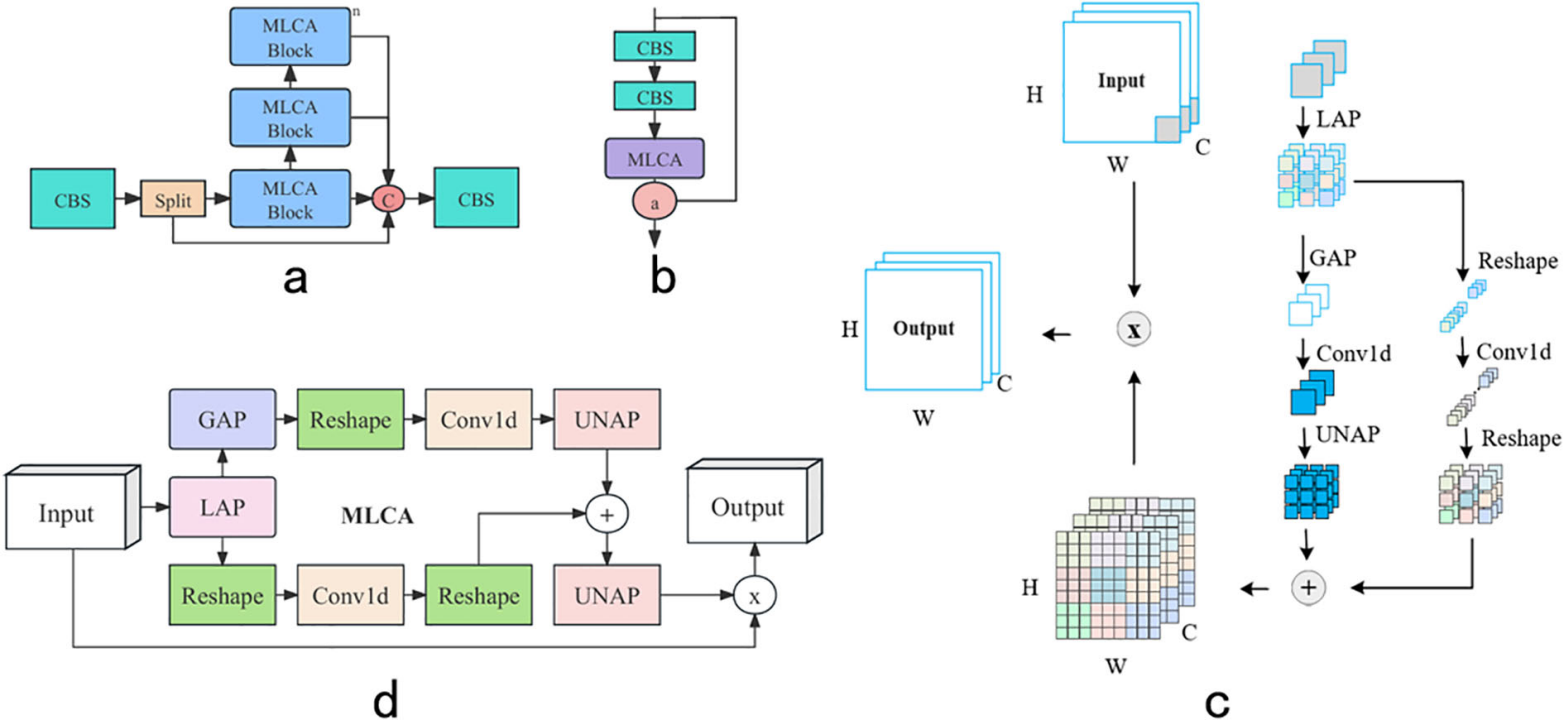
CM network structure diagram: **(a)** CM, **(b)** MLCA-Block, **(c)** MLCA attention schematic, **(d)** MLCA attention network structure diagram.

To address these issues, a new CM module was designed to replace the C2f module by introducing the MLCA (Mixed Local Channel Attention) mechanism in the bottleneck layer of the C2f module, as shown in **Figures 5a, b**. By considering channel and spatial attention at both local and global levels, the CM module significantly enhanced the capture of key features with minimal parameters, providing more diverse and rich feature representations and improving understanding and processing capabilities for complex scenarios, which resulted in a significant performance improvement in object detection tasks.

The excellent performance of the CM module benefited from the integration of the MLCA mechanism, illustrated in **Figures 5c, d**. MLCA was an efficient and lightweight attention mechanism that significantly improved object detection accuracy with only a small increase in parameters. It addressed the problems of traditional channel attention mechanisms that focused solely on one-dimensional weights while ignoring spatial information, as well as the high computational cost associated with spatial attention mechanisms. MLCA integrated channel and spatial information to balance model complexity and performance. The basic structure included Local Average Pooling (LAP) and Global Average Pooling (GAP). By performing 1D convolution on pooled features, rearranging and combining them with original features, and strengthening useful features, MLCA maintained computational efficiency while enhancing feature capture capabilities, achieving significant improvements in accuracy.

#### 2.3.4 Deep supervision auxiliary head

The traditional single-head network model, such as YOLOv8n, exhibited several shortcomings. Firstly, the model could not fully utilize the rich features of the middle layer if it relied solely on the final output for error feedback, which hindered the ability of this model to perform deep learning and feature refinement, causing it to fail to exploit its potential fully. Secondly, in situations where the target was small, occluded, or complex, such as the center leaf areas of corn, single-head network structures faced the problem of insufficient recall, meaning the model might not effectively identify or detect all relevant instances, especially in complex scenarios.

**Figure 6:**
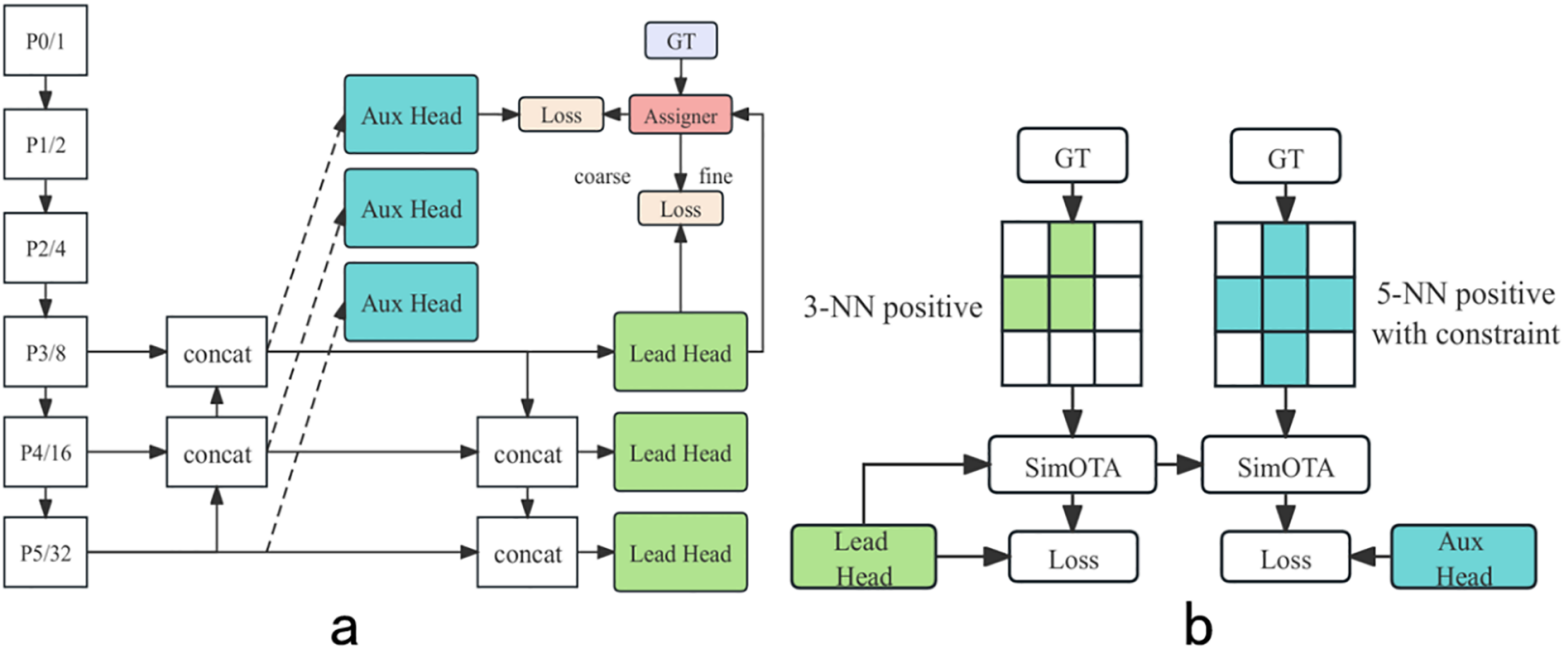
**(a)** Aux head structure diagram. **(b)** Coarse-to-fine lead head guided label assigner diagram.

To address the challenges brought about by the shortcomings of the standard YOLOv8 detection head, a method was proposed that combined an Auxiliary Head with the standard YOLOv8 detection head (Lead Head) (Wang et al., 2023). The improved detection module was shown in **Figure 6a**. The Aux Head was designed based on a deep supervision strategy, aiming to provide additional feature supervision through the auxiliary training head to optimize the capture of scale and hierarchical features. This design allowed the model to capture feature information of different scales and levels during the training process and use this information to optimize training results.

Specifically, the Aux Head combined the weights of the shallow network, auxiliary loss, and the final detection result with the real label to generate soft labels. These soft labels utilized finer-grained labels when training the Lead Head and coarser labels when training the Auxiliary Head, as shown in **Figure 6b**. This design enabled the Auxiliary Head to select additional cells as positive samples, thereby relaxing the restrictions on the potential area of positive samples and improving the recall rate.

In addition, the Auxiliary Head adopted the SimOTA algorithm (Ge, 2021; Ge et al., 2021), inheriting the idea of OTA to improve the allocation process of real samples. SimOTA considered the Intersection of Union (IoU) and classification results, further enhancing the performance. After introducing the Aux Head, the model overcame the detection challenges caused by the small size, overlapping, or high complexity of the targets in the center leaf areas of corn, achieving better training results.

### 2.4 Deployment on Jetson Xavier NX

To achieve real-time detection on resource-constrained edge devices in smart agriculture, the GMA-YOLOv8 model was deployed on the Jetson Xavier NX, eliminating delays associated with server communication. This embedded device was installed on unmanned spraying vehicles or robotic systems, functioning independently of network connections. The deployment process involved preparing a test dataset, importing and converting the trained model, compiling necessary libraries, and conducting rigorous performance evaluations. The system captured video streams using the DF-100 monocular camera, processed them with the GMA-YOLOv8 model, and displayed detection results in real-time on a connected monitor. Additionally, the ASDS algorithm was deployed on the Jetson Xavier NX to perform post-processing on YOLO outputs, with integrated signals communicated via a 232 serial port to the STM32 microcontroller. This approach significantly enhanced the reliability and feasibility of automated sustainable operations for unmanned spraying vehicles.

### 2.5 Experimental platform, environment, and parameter settings

#### 2.5.1 Model training platform

The experimental setup for this study was configured as follows: The deep learning model was trained and evaluated on an NVIDIA GeForce RTX 3090 GPU hosted on AutoDL platform. The experimental code was developed using Python 3.10.14, PyTorch 2.2.2+cu121, and torchvision 0.17.2+cu121.

#### 2.5.2 Training parameters

The improved networks were trained without using the pre-trained model yolov8n.pt. The learning rate (lr0) was set to 0.01, with the final learning rate (lrf) also at 0.01. Momentum was established at 0.937, and weight decay was configured to 0.0005. Warmup epochs were set to 3.0, with warmup momentum adjusted to 0.8 and warmup bias learning rate defined as 0.1. The number of workers was set to 4, and the optimizer chosen was SGD, with mixed precision (amp) enabled. Intersection over Union (IoU) was set to 0.7, replacing the previously considered value of 0.65 for consistency in training parameters. The number of epochs was set to 300, and the image size was defined as 640. These configurations ensured a consistent training environment and optimized model performance.

The selection of these hyperparameters was based on multiple considerations. First, through a comprehensive grid search approach, learning rates ranging from 0.001 to 0.1 were tested, where 0.01 demonstrated the optimal balance between convergence speed and stability. The momentum value of 0.937 and weight decay of 0.0005 were initially referenced from the successful practices in YOLOv8, and their effectiveness in this specific task was confirmed through experimentation. Additionally, ablation studies on these key parameters were conducted, which showed that this combination achieved the best trade-off between detection accuracy and training efficiency for corn leaf detection. Furthermore, these parameters also aligned well with the characteristics of the relatively small dataset, preventing overfitting while ensuring effective feature learning.

#### 2.5.3 Embedded development platform Jetson configuration

In this study, the configuration parameters of the Jetson embedded device were as follows: the NVIDIA Jetson Xavier NX Developer Kit operated on the Ubuntu 20.04 LTS (focal) platform. The system was powered by the Tegra194 SoC, featuring a CUDA architecture of 7.2. The software environment included PyTorch version 2.0.0, optimized for the NVIDIA Jetpack 5.1.1 framework, which provided enhanced performance for deep learning applications. Additionally, Torchvision version 0.15.1a0 was employed, ensuring compatibility with the latest features and improvements. The CUDA toolkit version 11.4.315 and cuDNN version 8.6.0.166 were also integrated, facilitating efficient GPU acceleration for both training and inference tasks. This setup was designed to leverage the powerful capabilities of the Jetson platform for advanced computer vision applications.

### 2.6 Model evaluation metrics

#### 2.6.1 Network model evaluation metrics

This study utilized a variety of metrics to measure improvements in model accuracy, including Precision (Equation 9), which represented the proportion of actual positives among predicted positives, and Recall (Equation 10), which indicated the proportion of actual positives that were correctly predicted. Subsequently, the average precision AP (Equation 11) was computed for a single class. For the entire task, the mean average precision ( mAP ) (Equation 12)was obtained by averaging all AP s corresponding to all classes. Additionally, the mAP50 metric was introduced, which represented the mean average precision under the condition of Intersection over Union (IoU) of 0.5. The prediction results were categorized into four classifications: True Positive ( TP ), True Negative ( TN ), False Positive ( FP ), and False Negative ( FN ), to comprehensively evaluate the performance.


(9) $$P=\frac{TP}{TP+FP}$$

(10) $$R=\frac{TP}{TP+FN}$$

(11) $$AP=\frac{1}{m}\sum_{i=1}^{m}P_{i}=\frac{1}{m}\cdot P_{1}+\frac{1}{m}\cdot P_{2}+\cdots +\frac{1}{m}\cdot P_{m}=\int^{}P\left(R\right)dR$$

(12) $$mAP=\frac{1}{C}\sum_{j=1}^{C}{AP}_{j}$$

To assess the complexity of the model, three key indicators were employed: the number of floating-point operations (FLOPs), the number of model parameters (Parameters), and the storage size of the model (Size). FLOPs measured the amount of computation required to execute the model, while the number of parameters reflected the complexity of the structure, and the storage size related to the convenience of model storage and deployment. During testing, all latency measurements were uniformly conducted on an NVIDIA RTX 3090 GPU to minimize performance variations that might occur on laptops. The results were obtained by calculating the average latency from three independent trials for each condition. The mean latency was determined to represent the central tendency of the measurements. Additionally, the standard deviation was calculated to assess the variability of the latency values across the trials. The final results are presented in the format of “Mean ± Standard Deviation” to provide a clear understanding of both the average performance and its consistency. These performance indicators offered a basis for a comprehensive understanding and optimization of the performance on edge devices.

#### 2.6.2 Evaluation metrics for the medication delivery system

To assess the effectiveness and reliability of the medication delivery system, the following evaluation metrics were established: Delivery Rate: The delivery rate measured the ratio of the number of plants that successfully received solid pesticide granules at the target areas to the total number of tested plants, as shown in Equation 13. $N_{\text{success}}$ represented the number of successful deliveries, and $N_{\text{total}}$ represented the total number of tested plants.

Detection Accuracy: The detection accuracy evaluates the ability of the deep learning model to accurately identify center leaf areas. It is calculated as the ratio of correctly identified leaf areas to the total number of leaf areas, as shown in Equation 14. $N_{\text{correct}}$ represents the number of correctly identified leaf areas, and $N_{\text{leaf}}$ represents the total number of leaf areas.

Delivery Precision: The delivery precision assesses the ratio of successfully delivered pesticide granules to accurately identified areas. It is calculated as the number of accurate deliveries to the number of identified target areas, as shown in Equation 15. $N_{\text{accurate}}$ represents the number of accurate deliveries, and $N_{\text{identified}}$ represents the number of identified target areas.


(13) $$Delivery\;Rate=\left(\frac{N_{\text{success}}}{N_{\text{total}}}\right)\times 100\%$$

(14) $$Detection\;Accuracy=\left(\frac{N_{\text{correct}}}{N_{\text{leaf}}}\right)\times 100\%$$

(15) $$Delivery\;Precision=\left(\frac{N_{\text{accurate}}}{N_{\text{identified}}}\right)\times 100\%$$

## 3 Results

This study conducted a comprehensive evaluation of network performance by presenting and analyzing results from computer-based training environment and the Jetson embedded platform. An in-depth exploration of the effectiveness of the network improvements was provided. Furthermore, the study examined how these enhancements impacted the functionality of the network across various application scenarios. Sections 3.1 to 3.4 focused on performance metrics on GPU platforms, while Section 3.5 was dedicated to the performance outcomes from field tests conducted on the Jetson embedded platform.

### 3.1 Comparative analysis of different backbone feature extraction networks

This study aimed to enhance the performance of YOLOv8n by integrating diverse architectural backbones, with comparative results presented in **Table 1**. The results revealed a consistent trade-off among model accuracy, size, and inference speed across various configurations.

**Table 1:** Comparative performance of different backbones applied in YOLOv8n.

Model	Dataset	Precision	Recall	mAP0.5 (Val)	Params (M)	FLOPs (G)	Size (MB)	Latency (MS)
V8n	D1	0.915	0.896	0.929	3.0	8.1	6.0	07.2 ± 0.6
	D2	0.867	0.805	0.883				
V8n+FasterNet	D1	0.852	0.886	0.896	4.1	10.7	8.2	08.6 ± 0.7
	D2	0.844	0.822	0.878				
V8n+GhostNet	D1	0.856	0.867	0.910	2.0	5.5	4.2	12.0 ± 0.1
	D2	0.862	0.794	0.876				
V8n+GhostNetV2	D1	0.854	0.896	0.925	3.0	7.2	6.3	11.4 ± 0.8
	D2	0.863	0.831	0.893				
V8n+MobileNetV2	D1	0.795	0.774	0.832	2.3	6.3	4.7	09.5 ± 0.5
	D2	0.843	0.809	0.872				
V8n+MobileNetV3	D1	0.824	0.85	0.827	2.2	5.4	4.5	09.9 ± 0.2
	D2	0.854	0.791	0.887				
V8n+EfficientViT	D1	0.884	0.886	0.924	4,0	9.4	8.4	22.9 ± 0.6
	D2	0.844	0.778	0.856				
V8n+HGNetV2	D1	0.854	0.851	0.894	2.3	7.0	5.0	07.8 ± 0.4
	D2	0.876	0.804	0.891				
V8n+RepHGNetV2	D1	0.820	0.879	0.898	2.3	6.9	4.8	08.0 ± 0.9
	D2	0.846	0.780	0.865				
V8n+GhostHGNetV2	D1	0.857	0.908	0.918	2.3	6.8	4.7	08.0 ± 0.1
	D2	0.859	0.813	0.888				
**V8n+GHG2S**	**D1**	**0.901**	**0.872**	**0.920**	**2.3**	**6.8**	**4.9**	**08.0 ± 0.3**
	**D2**	**0.855**	**0.827**	**0.888**				

In order to ensure that different feature extraction networks applied after YOLOv8n had model sizes on the same order of magnitude, the following specific models were selected: FasterNet_t0, GhostNet_050, GhostNetV2_100, MobileNetV2_050, MobileNetV3_small_050, and EfficientViT_M0. The Latency values were measured with the batch size set to 1. The bolded text indicates the best-performing network architecture.

While the baseline YOLOv8n demonstrated robust initial performance (mAP: 0.929 on Dataset D1, 0.883 on Dataset D2), attempts to improve specific aspects yielded varied outcomes. For instance, models such as GhostNet and GhostNetV2 (Tang et al., 2022) achieved notable detection performance, albeit at the expense of slightly increased inference latency (12.0 ± 0.1 MS and 11.4 ± 0.8 MS, respectively). Conversely, the MobileNet series and FasterNet (Chen et al., 2023) prioritized inference velocity, which generally led to a reduction in detection accuracy. EfficientViT (Liu et al., 2023) exhibited competitive accuracy on one dataset but displayed a marked decrease on another, coupled with slower processing speeds. The HGNet series, however, presented a more balanced approach, maintaining efficiency and consistent performance. Within this series, the enhanced GhostHGNetV2 and GHG2S variants were particularly prominent, aligning with the conclusions of Huang et al. (2024) and Yan et al. (2024), distinguished by their minimal parameter count (2.3M) and FLOPs (6.8G).

Ultimately, YOLOv8n+GHG2S was identified as the optimal configuration. It achieved an exceptional balance of high detection precision (D1: 0.920 mAP, D2: 0.888 mAP) and rapid inference speed (08.0 ± 0.3 MS). This renders YOLOv8n+GHG2S particularly well-suited for demanding edge computing applications in precision agriculture, effectively mitigating the typical performance degradation associated with lightweight backbone designs.

### 3.2 Ablation analysis of the impact of attention application methods on deep model performance

#### 3.2.1 Attention contrast experiments in main network

**Figure 7:**
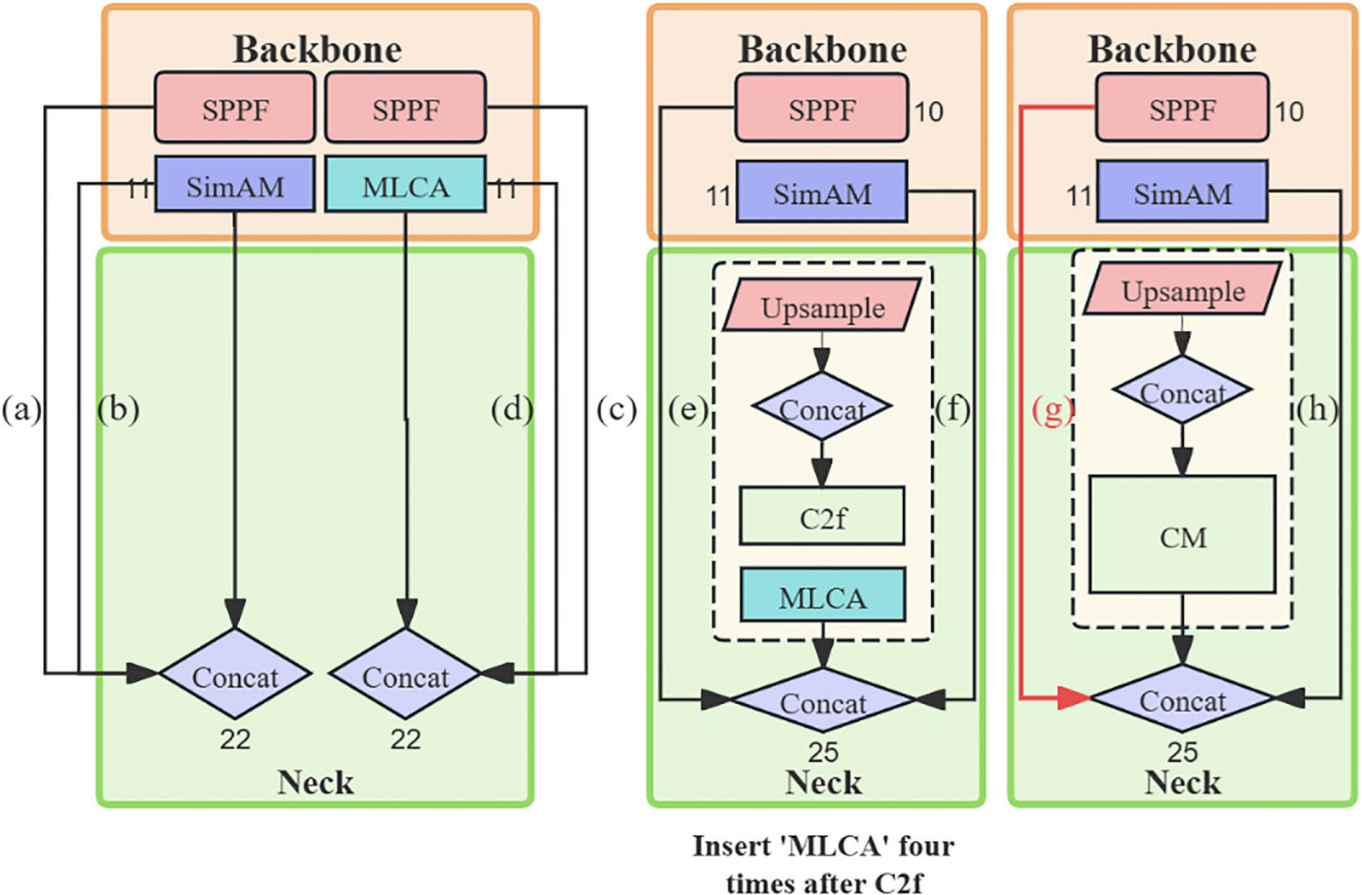
Different applications of attention. **(a, b)** two structures using SimAM attention, **(c, d)**, two structures using MLCA, **(e, f)**, two structures using SimAM and MLCA, **(g, h)**, two structures using SimAM and CM.

To overcome the limitations of single-level feature extraction in network architectures and compensate for performance loss incurred by lightweight backbone networks, this study introduced the three-dimensional attention mechanism SIMAM and the mixed channel-space attention mechanism MLCA. Ablation experiments were conducted focusing on the critical p5 layer (methodologies illustrated in **Figure 7**) to optimize detection performance.

Results presented in **Table 2** elucidate that **Figure 7b**, employing SIMAM, achieved higher accuracy on both datasets compared to **Figure 7a**, while MLCA in **Figure 7c** demonstrated stronger detection capabilities than **Figure 7d**. This discrepancy highlights the differential information processing abilities of SIMAM and MLCA. SIMAM enhanced the sensitivity to three-dimensional cues, whereas MLCA prioritized the augmentation and integration of salient features along with contextual information within the feature map. Furthermore, the design of scheme (c) significantly mitigated the risk of excessive feature amplification, thereby improving detection capabilities.

**Table 2:** Ablation analysis of the impact of attention application methods on deep model performance.

Model	Dataset	Precision	Recall	mAP0.5 (Val)	Params (M)	FLOPs (G)	Size (MB)	Latency (MS)
GY-S_10 (a)	D1	0.857	0.926	0.926	2.3	6.8	6.2	8.5 ± 0.2
	D2	0.882	0.795	0.880				
GY -S_11 (b)	D1	0.893	0.903	0.934	2.3	6.8	6.2	8.5 ± 0.6
	D2	0.870	0.819	0.896				
GY -M_10 (c)	D1	0.875	0.900	0.933	2.3	6.8	6.2	9.0 ± 0.1
	D2	0.888	0.821	0.891				
GY -M_11 (d)	D1	0.867	0.899	0.916	2.3	6.8	6.2	8.4 ± 0.6
	D2	0.887	0.807	0.897				
GY -S_10+MLCA (e)	D1	0.864	0.900	0.904	2.3	6.8	6.2	9.5 ± 0.4
	D2	0.868	0.812	0.890				
GY -S_11+MLCA (f)	D1	0.889	0.908	0.932	2.3	6.8	6.2	8.9 ± 0.7
	D2	0.859	0.816	0.887				
**GY -S_10+CM (g)**	**D1**	**0.912**	**0.925**	**0.945**	**2.3**	**6.8**	**6.2**	**9.1 ± 0.3**
	**D2**	**0.899**	**0.805**	**0.901**				
GY -S_11+CM (h)	D1	0.9	0.867	0.929	2.3	6.8	6.2	9.1 ± 0.6
	D2	0.878	0.806	0.888				

In this table, “GY” stands for GMA-YOLOv8, “S” stands for SimAM attention, “M” stands for MLCA attention. The bolded text indicates the best-performing network architecture.

These results confirm the efficacy of SimAM in optimizing feature extraction of GHG2S backbone. Inspired by research of Huang et al. (2022) on selective connection mechanisms, this study further explored optimal attention mechanism configuration strategies. Results demonstrate that scheme (g) achieved the best overall performance across both datasets, strongly validating the superiority of strategically fusing different attention mechanisms, rendering it an ideal choice for high-precision, high-efficiency agricultural edge detection.

#### 3.2.2 Attention contrast experiments in neck network

Based on the design of incorporating SIMAM attention into the GHG2S backbone, supplementary experiments were conducted to optimize the feature-capturing deficiencies introduced by the original C2f structure. These experiments explored the efficacy of attention mechanisms within the neck network, as depicted in **Figure 7e–h**. Initial attempts to integrate MLCA into the neck network and increase its depth led to decreased performance, as shown in **Figure 7e, f**. However, strategically applying attention mechanisms within the bottleneck of the C2F structure, along with the development of the CM module, boosted performance to a mean average precision (mAP) of 94.5%, as illustrated in **Figure 7g**, while maintaining an acceptable inference velocity.

The success of the CM module lay in its ability to alleviate potential issues by directing focus towards essential features and optimizing the processing of multi-scale information. Consequently, it dynamically enhanced critical features to improve computational efficiency and achieve a balance between performance and speed.

#### 3.2.3 The synergistic integration of SIMAM and MLCA mechanisms

In essence, the juxtaposition and ablation studies conducted indicated that the synergistic application of SimAM and the MLCA-bearing CM module to the network structure (scheme g) facilitated optimal feature extraction and surpassed the outcomes achievable through their isolated application. This synergy not only addressed the limitations inherent in the singular applications of each mechanism but also fostered their mutual enhancement, thereby augmenting the capability of this network to extract complex features and amalgamate contextual data. This, in turn, markedly boosted detection accuracy, substantiating the premise that the proposed integration of SimAM and MLCA attention mechanisms could effectively resolve the challenges associated with single-level feature extraction.

#### 3.2.4 Backbone and attention visualization analysis

Gradient-weighted Class Activation Mapping (Grad-CAM) (Selvaraju et al., 2020) was employed to generate heatmaps visualizing the center leaf areas, where GHG2 represents GhostHGNetv2. The heatmaps provided intuitive visualization of the regions in feature maps that attracted the attention. Through backpropagation of the output class confidence, gradient values were calculated, with higher values displayed in deeper red and lower values in deeper blue. The heatmap generation utilized the following parameters: method: Grad-CAM; layers: [10, 12, 14, 16, 18]; backward type: ‘class’; confidence threshold: 0.2; ratio: 0.02; show box: False; renormalize: True.

**Figure 8:**
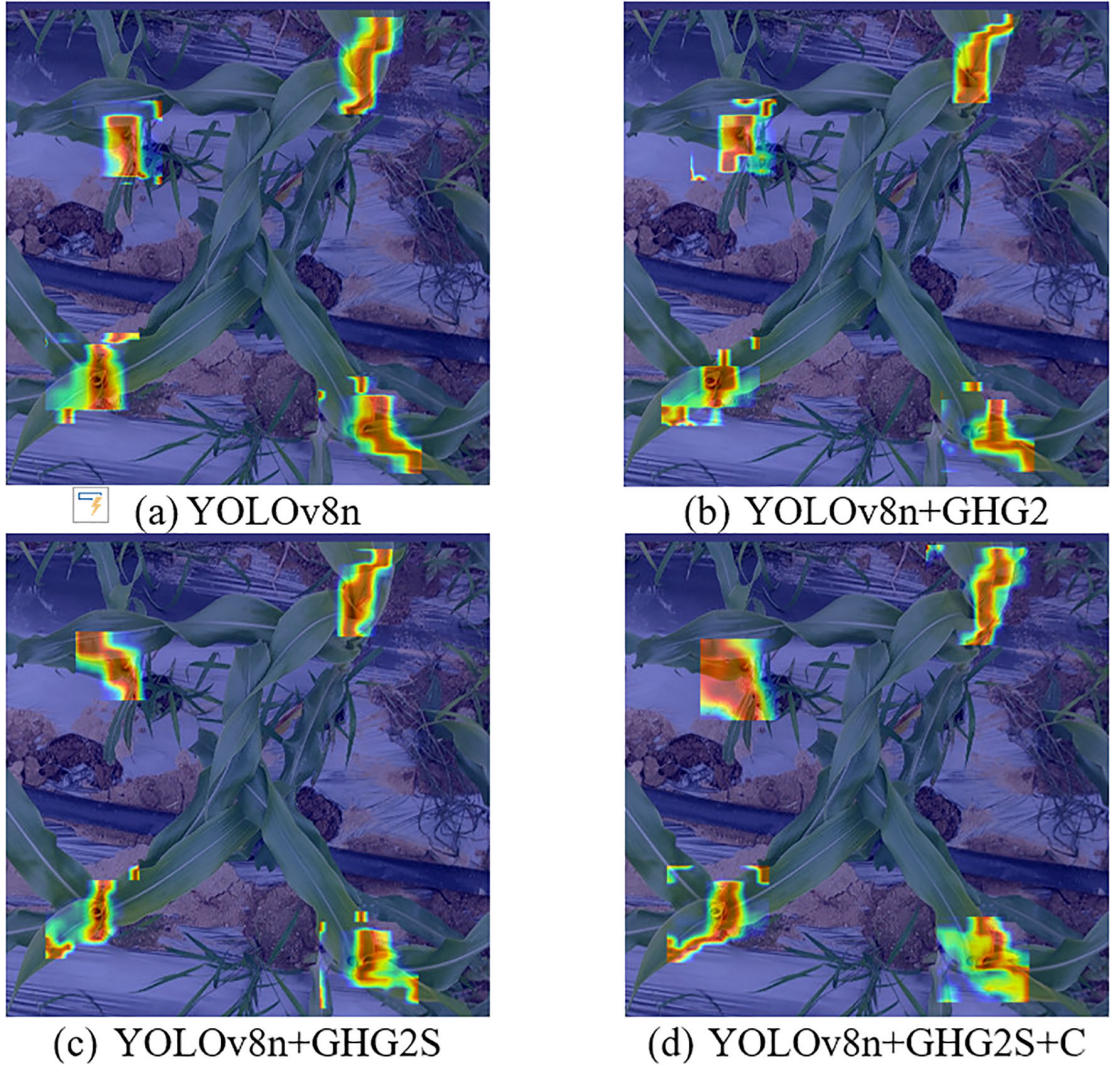
Grad-CAM visualization heatmaps for different backbone and attention module configurations: **(a)** YOLOv8n, **(b)** YOLOv8n+GHG2, **(c)** YOLOv8n+GHG2S, **(d)** YOLOv8n+GHG2S+C.

- YOLOv8n: Small, concentrated areas of high activation on corn leaves, indicating focus on specific, localized features. (**Figure 8a**)
- YOLOv8n+GHG2: Slight shift in attention patterns with GHG2 backbone, showing both diffuse and sharp focus areas, suggesting altered feature extraction. (**Figure 8b**)
- YOLOv8n+GHG2S: More coherent and broader activation areas with SimAM module, implying improved feature integration and attention to larger contextual regions. (**Figure 8c**)
- YOLOv8n+GHG2S+C: Most comprehensive attention distribution with CM, showing larger, intense activation areas across corn structures. This suggests enhanced capture of holistic features and contextual information, potentially improving detection in complex backgrounds. (**Figure 8d**)

The heatmap visualization intuitively validated the effectiveness of the proposed improvements.

### 3.3 Albation study

This study, based on YOLOv8n, achieved an optimal balance between model lightness and performance efficiency through three progressive improvement strategies, with ablation study results detailed in **Table 3**.

**Table 3:** Ablation study results: model performance and efficiency metrics of different configurations.

Method	Dataset	Precision	Recall	mAP0.5 (Val)	Params (M)	FLOPs (G)	Size (MB)	Latency (MS)
YOLOv8n	D1	0.915	0.896	0.929	3.0	8.1	6.0	07.2 ± 0.6
	D2	0.867	0.805	0.883				
YOLOv8n+G	D1	0.901	0.872	0.920	2.3	6.8	4.9	08.0 ± 0.3
	D2	0.855	0.827	0.888				
YOLOv8n+G+C	D1	0.916	0.912	0.935	2.3	6.9	4.7	9.0 ± 0.5
	D2	0.869	0.828	0.890				
**YOLOv8n+G+C+A**	**D1**	**0.912**	**0.925**	**0.945**	**2.3**	**6.8**	**6.2**	**9.1 ± 0.3**
	**D2**	**0.899**	**0.805**	**0.901**				

In this table, “G” stands for GHG2S, “C” stands for CM, “A” stands for Aux Head. The Latency values were all measured with the batch size set to 1. The bolded text indicates the best-performing network architecture.

Firstly, by introducing the GHG2S backbone network (YOLOv8n+G), model complexity was significantly reduced, with parameters, FLOPs, and model size substantially decreased (2.3M parameters, 6.8G FLOPs, 4.9MB size), while maintaining high mAP on both datasets (D1: 0.920, D2: 0.888). GHG2S, leveraging hierarchical feature extraction of HGNetV2 and the SimAM attention mechanism, enabled efficient feature extraction without sacrificing performance. Subsequently, the innovative CM design (YOLOv8n+G+C) further enhanced model performance (D1 mAP: 0.935, D2 mAP: 0.890) by integrating channel and spatial attention mechanisms to optimize feature integration, with only a minimal increase in computational load. Finally, the integration of the AuxHead component (YOLOv8n+G+C+A) led to the highest mAP scores (D1: 0.945, D2: 0.901) and a significantly improved recall rate, primarily attributable to the enhanced training quality provided by deep supervision.

In summary, through these optimization strategies and innovative designs specifically targeting the center leaf areas of corn, the proposed model achieved excellent detection performance while maintaining its lightweight nature (2.3M parameters, 6.8G FLOPs) and computational efficiency. The final configuration balanced model compression and inference speed, demonstrating a latency of 9.1 ± 0.3 MS. These consistent performance improvements validate the capability to significantly enhance efficiency and efficacy in computer vision tasks, particularly for edge computing scenarios like those involving agricultural robots.

### 3.4 Comparison of state-of-the-art methods

**Figure 9:**
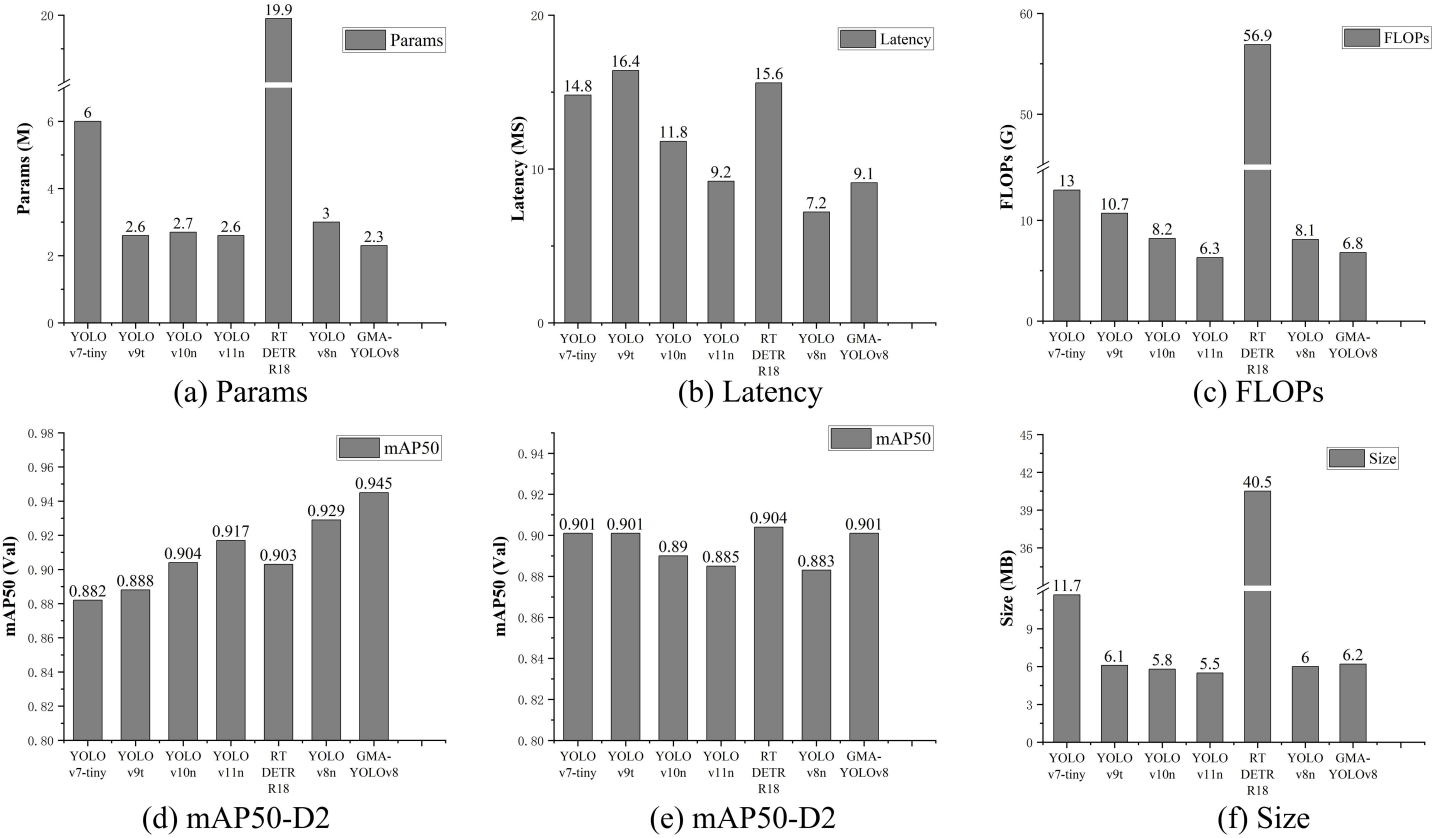
Comparison of experimental results of different models: **(a)** Params, **(b)** Latency, **(c)** FLOPs, **(d)** mAP50-D1, **(e)** mAP50-D2, **(f)** Size.

The comparative analysis between the advanced network architecture and conventional detection algorithms demonstrated that the enhanced algorithm achieved a superior mAP50 score of 0.945 on the first dataset (D1), surpassing all other competing methods. Although the performance gap is 0.3 mAP50 points compared with RT-DETR-R18 on the second dataset (D2), the proposed GMA-YOLOv8 algorithm shows comprehensive advantages in lightweight implementation. The architectural efficiency was particularly noteworthy, as the model was developed with approximately one-tenth of the parameter volume required by conventional counterparts, while simultaneously achieving twice the operational speed of comparable algorithms. This systematic evaluation, which encompassed both detection precision and computational efficiency, confirmed the effectiveness and technical superiority of the algorithm. Complete performance metrics for all compared models were systematically documented in **Table 4** and **Figure 9**.

**Table 4:** Comparison of experimental results of different models.

Model	mAP0.5(Val)		Params (M)	FLOPs (G)	Size (MB)	Latency (MS)
	Dataset 1	Dataset 2				
YOLOv7-tiny	0.882	0.901	6.0	13.0	11.7	14.8 ± 0.3
YOLOv9t	0.903	0.901	2.6	10.7	6.1	16.4 ± 0.8
YOLOv10n	0.904	0.890	2.7	8.2	5.8	11.8 ± 0.1
YOLOv11n	0.917	0.885	2.6	6.3	5.5	09.2 ± 0.6
RT-DETR-R18	0.903	0.904	19.9	56.9	40.5	15.6 ± 0.4
YOLOv8n	0.929	0.883	3.0	8.1	6.0	07.2 ± 0.6
**GMA-YOLOv8**	**0.945**	**0.901**	**2.3**	**6.8**	**6.2**	**09.1 ± 0.3**

The Latency values were all measured with the batch size set to 1. The bolded text indicates the best-performing network architecture.

**Figure 10:**
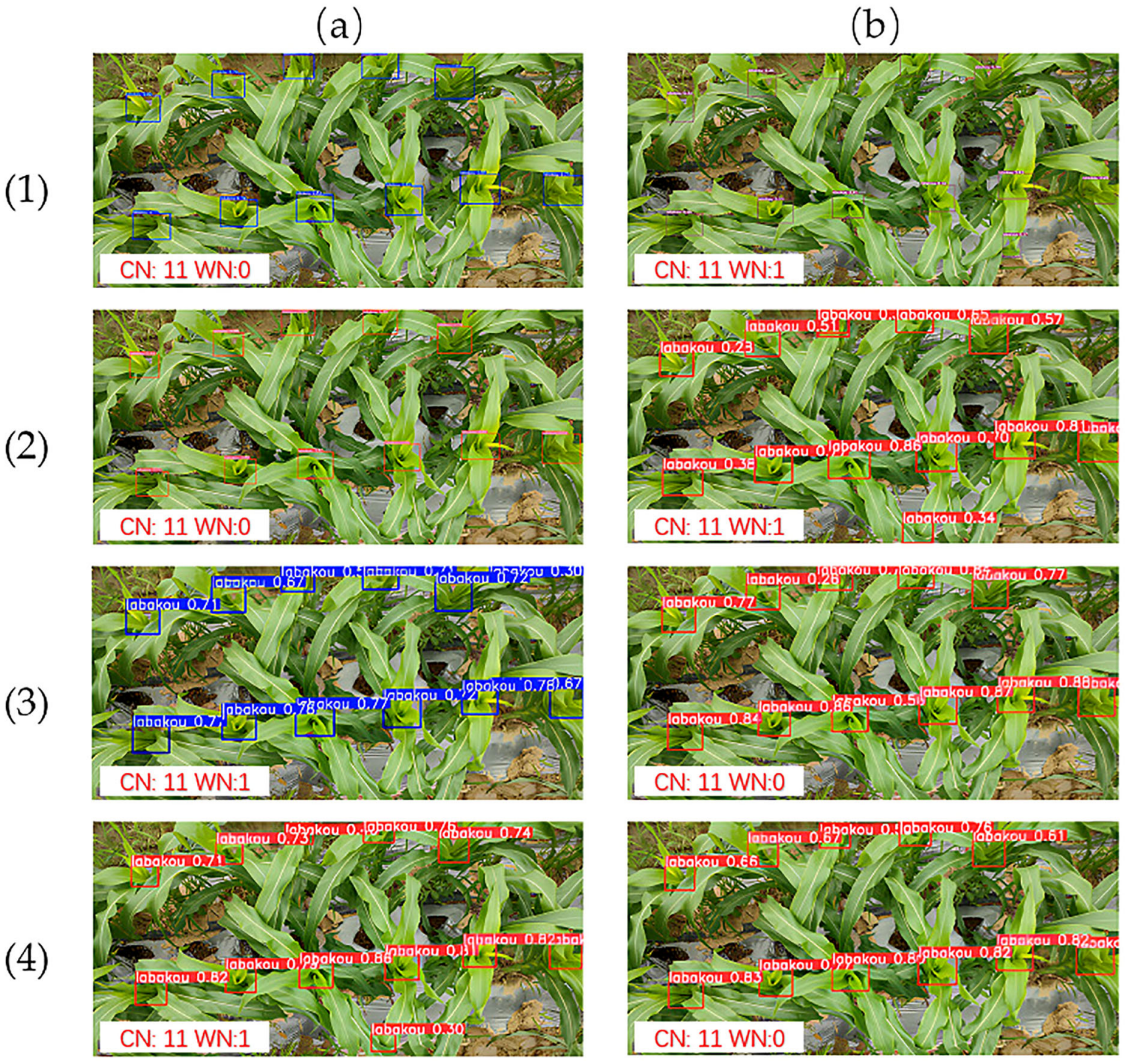
Object detection model comparison. **(a1)** YOLOv5n, **(b1)** YOLOv7-tiny, **(a2)** YOLOv9t, **(b2)** YOLOv10n, **(a3)** YOLOv11n, **(b3)** RT-DETR-R18, **(a4)** YOLOv8n, and **(b4)** GMA-YOLO. CN denotes correct number and WN denotes wrong number.

**Figure 10** presents the comparison of detection performance for various models.

Analysis of these results reveals a broad performance spectrum. YOLOv7-tiny showed the lowest actual detection confidence rate, while YOLOv10n demonstrated strong detection for central targets but lower confidence for peripheral ones. RT-DETR-R18 achieved high overall confidence rates, though it risked low confidence (0.26) for slightly occluded targets. YOLOv5n, YOLOv8n, YOLOv9t, and GMA-YOLO consistently showed progressively improving performance. Notably, all lightweight YOLO variants (YOLOv7-tiny, YOLOv10n, YOLOv11n, and YOLOv8n) demonstrated false detections with low confidence scores, potentially due to model overfitting issues. Ultimately, GMA-YOLO achieved the best overall detection performance among the compared models. This comparative analysis provides an intuitive visualization of each strengths and limitations in object detection tasks from a top-down perspective.

### 3.5 GMA-YOLO performance across planting densities and growth stages

**Figure 11:**
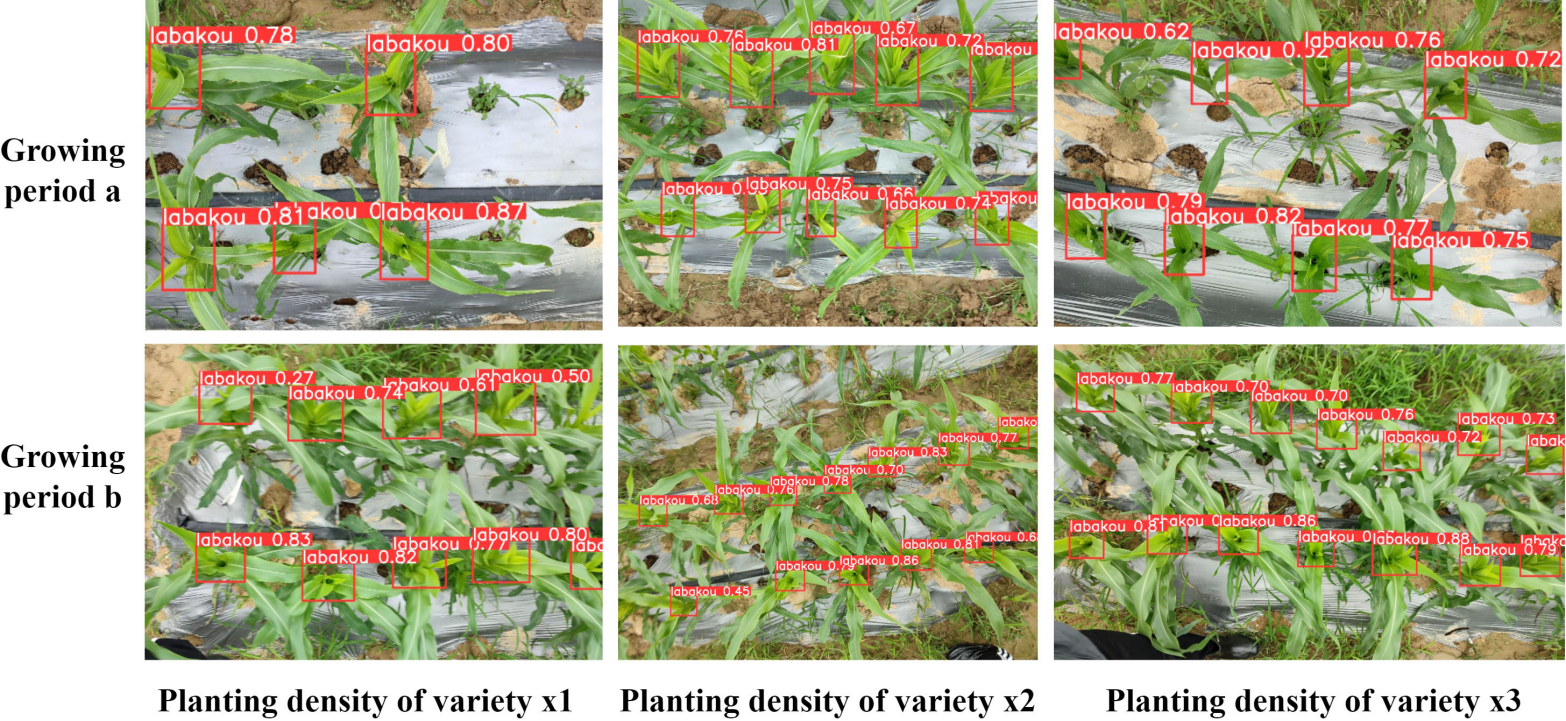
GMA-YOLO detection of corn at different growth stages and planting densities.

To comprehensively evaluate and demonstrate the GMA-YOLO detection performance and generalization capability in real agricultural environments, this study conducted in-depth detection experiments focusing on corn plants under varying planting densities (e.g., sparse vs. dense cultivation) and at different crop developmental stages (ranging from seedling to maturity, involving dynamic changes in plant size, leaf morphology, and mutual occlusion levels). These conditions were designed to fully simulate the complexity and diversity of real field environments, thereby verifying the robustness and adaptability in complex, dynamic settings. The detection results are shown in **Figure 11**.

### 3.6 Field performance

In real corn field conditions, a systematic test of the pesticide application system prototype was conducted to verify its effectiveness. The main debugging equipment included an NVIDIA Jetson NX embedded device, a 640 × 480 resolution display, a keyboard, and a mouse, which were used to adjust the rectangle size in the ASDS algorithm and the rotation speed parameters of the funnel motor. During the testing process, the application system was mounted on a mobile platform, with the camera fixed approximately 0.5 meters above the corn plants. Influenced by the best practices of YOLO, the experiments were conducted under the conditions of conf_thres=0.25, iou_thres=0.45. The test results demonstrated that the sprayer prototype could effectively detect the center leaf areas of the corn and apply pesticides via the rotating motor. In the field experiments, all groups except the first (45 plants) were tested on 50 corn plants each. The experimental results, summarized as Mean ± Standard Deviation, are presented in Table X. Specifically, the Delivery Rate was 84.1 ± 3.3%, Detection Accuracy was 91.3 ± 1.9%, and Delivery Precision was 92.2 ± 2.9%. The test results are shown in **Table 5**. This section provided a comprehensive overview of the field testing results, highlighting the performance of the application system and the challenges faced, aiming to offer reference points for future research.

**Table 5:** Field test results.

$N_{\text{total}}$	$N_{\text{identified}}$	$N_{\text{success}}$	Delivery rate	Detection accuracy	Delivery precision	$N_{\text{total}}$
1	45	42	40	88.9	93.3	95.2
2	50	45	43	86.0	90.0	95.6
3	50	46	42	84.0	92.0	91.3
4	50	44	41	82.0	88.0	93.2
5	50	46	40	80.0	92.0	87.0
6	50	47	44	88.0	94.0	93.6
7	50	45	40	80.0	90.0	88.9
8	50	46	43	86.0	92.0	93.5
9	50	45	41	82.0	90.0	91.1
Total	445	406	374	84.0	91.2	92.1
M ± SD				84.1 ± 3.3	91.3 ± 1.9	92.2 ± 2.9

It should be specifically noted that the field validation experiments were exclusively conducted under Dataset D1 conditions. While Dataset D2 was subsequently incorporated for extended algorithm validation, its field testing was constrained by seasonal agricultural cycles and equipment availability limitations. The laboratory-based comparative analysis on D2 demonstrated comparable performance trends to those observed in D1 field trials, with detailed metrics presented in Section 3.3.

#### 3.6.1 Deep model field test results

To evaluate the accuracy and applicability of the designed deep learning model, field trials were conducted. During these tests, video data output from the portable display was recorded in real-time for subsequent statistical analysis. Out of 445 corn plants tested, the GMA-YOLOv8 deep learning model successfully identified 406 plants, failing to detect 39. This resulted in a detection accuracy of 91.3 ± 1.9%. Additionally, the YOLO model achieved a frame rate of approximately 30 fps on the Jetson device.

#### 3.6.2 Medication delivery system results

To assess the effectiveness and reliability of the pesticide application system, a thorough inspection and documentation of the treated corn plants were conducted. Out of the 445 plants, solid pesticide granules were successfully delivered to the tender leaf areas of 374 plants, with 71 plants not receiving treatment. This resulted in a delivery rate of 84.1 ± 3.3%.

#### 3.6.3 Analysis of unsuccessful deliveries

A detailed comparison of the test records revealed that 71 plants did not receive treatment. Of these, 39 were due to the deep learning model failing to accurately detect the tender leaf areas of the corn plants, and 32 were due to the application system not accurately delivering the pesticide granules to the corresponding areas. Specifically, the delivery precision was calculated based on the 374 accurate deliveries out of the 406 identified target areas, resulting in a delivery precision of approximately 92.2 ± 2.9%. Through this analysis, it was concluded that while the application system performed well in most cases, further optimization of the deep learning model and application mechanism is necessary to enhance overall detection and application accuracy.

#### 3.6.4 Field test results analysis and system performance evaluation

Field testing revealed 39 detection failures, primarily attributable to environmental complexity and model performance limitations. Detailed analysis showed that more than half of these cases failed due to spatial overlap of corn leaves causing partial occlusion of target areas, which exceeded the recognition capabilities of the vision model. The remaining cases likely resulted from multiple factors, including model precision limitations, insufficient environmental adaptability, and adverse imaging conditions, such as camera angle and motion blur induced by platform movement. Despite these challenges, the GMA-YOLOv8 model demonstrated a field detection accuracy of 91.3 ± 1.9%, providing clear directions for further optimization.

Regarding delivery precision, analysis identified 32 cases of inaccurate delivery. Benefiting from the synergistic design of the FCRF mechanical structure and ASDS algorithm, the system employs an asynchronous execution strategy for detection and delivery, effectively preventing the accumulation of mechanical errors. The delivery deviations were primarily attributed to timing mismatches caused by signal processing delays. Additionally, the significant terrain undulations in the experimental plot affected equipment stability. Notably, the trajectory of solid pesticide granules was minimally affected by wind forces, rendering this factor negligible. Overall, the FGA-Corn system achieved a delivery precision of 92.2 ± 2.9%, establishing a foundation for future applications in complex agricultural environments. Subsequent optimizations will focus on environmental factors, particularly terrain adaptability.

## 4 Discussion

This study introduced FGA-Corn, a vision-based precision pesticide application system specifically designed to target the center leaf areas of corn plants. The system integrated a simple yet efficient mechanical structure, an ASDS post-processing algorithm that synergistically combined deep learning with mechanical operation, and a lightweight, high-precision object detection model. Unlike existing systems that often sprayed over the entire target area despite incorporating visual algorithms (Hu et al., 2024; El Amraoui et al., 2024; Upadhyay et al., 2024), FGA-Corn innovatively shifted the spraying target to localized regions of the plant, providing a novel solution for high-precision pesticide application.

The proposed GMA-YOLO model demonstrated exceptional performance, being both lighter (with a 23.3% reduction in model size and a computational complexity of 6.8 GFLOPs) and more accurate (achieving mAP@0.5 scores of 94.5% (+1.6%) and 90.1% (+1.8%), respectively). Field test results further confirmed the reliability and effectiveness, showing a detection accuracy of 91.3 ± 1.9% for corn center leaves, a pesticide delivery rate of 84.1 ± 3.3%, and a delivery precision of 92.2 ± 2.9%.

Field tests in this study were primarily conducted using Dataset D1. Additionally, an independent corn growth stage dataset, D2, was collected and tested under laboratory conditions. Laboratory results indicated that the overall performance trends of D2 were consistent with those of D1, which validated the stability and generalizability of the proposed method.

The design of the FGA-Corn system drew inspiration from the morphological characteristics of corn plants, particularly the funnel-like structure of the upper plant canopy. This design facilitated the gravitational flow of applied pesticides to the target central area. However, the accuracy of this vision-based spraying method was highly dependent on visual information and presented challenges such as leaf occlusion. Two main approaches were considered to address this: optimizing the visual algorithm itself, or employing external equipment during field operations, such as devices designed to gently move leaves aside.

Beyond the V6-V8 growth stages, which were the primary focus of this study, the FGA-Corn system was also applicable to both early and late growth stages of corn. Its applicability was notably stronger in the late stages compared to early stages, as mature corn plants, similar to those in the V6-V8 period, possessed larger leaf areas, facilitating better gravitational movement of pesticide particles to the target region. While suitable for young corn, the smaller target area and leaf size in seedling plants might slightly increase the difficulty of pesticide application. For different growth stages, localized precision spraying could be achieved by adjusting parameters within the FGA-Corn system, such as the distance from the funnel to the corn tender leaf center and the rotation speed of the funnel. In practical applications, the main spraying period was concentrated during the early to mid-growth stages of corn plants.

Despite these significant achievements, this study still identified limitations and areas for future improvement. To enhance the understanding of the decision-making process of GMA-YOLO model, further exploration into model interpretability, utilizing visualization methods like CAM, was needed.

Furthermore, improving the generalization capability under varying environmental conditions was considered crucial. This could be approached through two primary avenues: first, from the data perspective, for example, by employing data augmentation strategies as utilized in this study or by expanding the dataset to cover diverse conditions; and second, through the assistance of external equipment, such as adding sunshades, rain shelters, and fill lights to maintain optimal and consistent light intensity during mornings, noons, evenings, and cloudy conditions. Given that actual spraying operations were typically avoided during rainfall to prevent pesticide runoff and muddy ground conditions, physically maintaining constant light intensity was deemed more efficient for practical engineering deployment.

Future research will also focus on optimizing mechanical structures to enhance flexibility and durability. To reduce system costs and optimize performance, studies on model quantization and pruning techniques will be conducted to further reduce computational load, and efforts will be made to deploy the system on affordable embedded platforms like Raspberry Pi or RK3588. While the system performed well in structured fields, challenges in complex terrains will be addressed in future work through improved mechanical design and extensive validation data collection. Additionally, research on the adaptability of the system to different crops and comprehensive environmental impact assessments are planned to ensure sustainable agricultural implementation.

## 5 Conclusion

This study addresses the need for precision pesticide application on corn center leaf areas by proposing the FGA-Corn system, which integrates an innovative mechanical structure, an intelligent decision algorithm, and an enhanced lightweight detection model. The system offers a novel solution for high-precision pesticide spraying and holds significant practical value for applying machine vision in precision agriculture. The key conclusions are as follows.

1. This study designed the FCRF mechanical structure and combined it with the ASDS decision algorithm to achieve precise spraying based on real-time visual perception. Field trials demonstrated a tender-leaf detection efficiency of 91.3 ± 1.9%, pesticide delivery efficiency of 84.1 ± 3.3%, and delivery precision of 92.2 ± 2.9%. Deployment on the Jetson Xavier NX platform confirmed the engineering feasibility of system in real agricultural environments.
2. This study developed a lightweight GMA-YOLOv8 model that, through architectural optimization, improved mAP from 92.9% to 94.5% on Dataset D1 and from 88.3% to 90.1% on Dataset D2 (average gain of 1.7%), while reducing computational load to 6.8 GFLOPs. Its stable cross-dataset performance validates both the effectiveness of algorithmic enhancements and strong generalization capability of this study, establishing a new technical benchmark for embedded agricultural vision systems.
3. This work shifts the pest and disease management paradigm from field-scale coverage to localized, per-plant targeting, constructing an automated precision spraying framework that synergizes vision algorithms with mechanical execution. This comprehensive solution mitigates agricultural pollution and food safety risks, holding important practical implications for sustainable agricultural development. While significant progress was made, future work will focus on improving detection robustness in varied environmental conditions, developing more adaptive mechanical structures for diverse terrains, optimizing model efficiency for low-power edge devices, and conducting broader field trials to assess system generality.

## Data Availability

The original contributions presented in the study are included in the article/supplementary material, further inquiries can be directed to the corresponding author/s.
